# Ultrasound-Assisted Mild Heating Treatment Improves the Emulsifying Properties of 11S Globulins

**DOI:** 10.3390/molecules25040875

**Published:** 2020-02-17

**Authors:** Linlin Liu, Jianhua Zeng, Bingyu Sun, Na Zhang, Yinyuan He, Yanguo Shi, Xiuqing Zhu

**Affiliations:** Key Laboratory of Grain Food and Comprehensive Processing of Grain Resource of Heilongjiang Province, Key Laboratory of Food Science and Engineering of Heilongjiang Province, College of Food Engineering, Harbin University of Commerce, Harbin 150076, China; keaiduolinlin@126.com (L.L.); foodleslie@163.com (J.Z.); sby0451@163.com (B.S.); foodzhangna@163.com (N.Z.); heyinyuan717@163.com (Y.H.)

**Keywords:** ultrasound, mild heating, structure, emulsifying property, glycinin

## Abstract

Ultrasonic technology is often used to modify proteins. Here, we investigated the effects of ultrasound alone or in combination with other heating methods on emulsifying properties and structure of glycinin (11S globulin). Structural alterations were assessed with Sodium dodecyl sulphate-polyacrylamide gel electrophoresis (SDS–PAGE), intrinsic fluorescence spectroscopy, ultraviolet (UV) absorption spectroscopy, and Fourier transform infrared (FTIR) spectroscopy. The size distribution and zeta-potential of 11S globulin were evaluated with a particle size analyzer. An SDS-PAGE analysis showed no remarkable changes in the primary structure of 11S globulin. Ultrasound treatment disrupted the 11S globulin aggregates into small particles with uniform size, narrowed their distribution and increased their surface charge density. Fluorescent spectroscopy and second-derivative UV spectroscopy revealed that ultrasound coupled with heating induced partial unfolding of 11S globulin, increasing its flexibility and hydrophobicity. FTIR further showed that the random coil and α-helix contents were higher while β-turn and β-sheet contents were lower in ultrasound combined with heating group compared to the control group. Consequently, the oil-water interface entirely distributed protein and reduced the surface tension. Moreover, ultrasound combined with heating at 60 °C increased the emulsifying activity index and emulsifying stability index of 11S globulins by 6.49-folds and 2.90-folds, respectively. These findings suggest that ultrasound combined with mild heating modifies the emulsification properties of 11S globulin.

## 1. Introduction

Proteins extracted from soybeans are essential ingredients for industrial food products such as beverages and nutraceuticals because they are affordable, with health benefits [1,2,3]. However, untreated soy proteins are not readily applicable because of limited surface-active properties (e.g., emulsifying property) and solubility. Moreover, in untreated state, soy proteins have quaternary and tertiary compact structures [3,4,5]. It is, therefore, imperative to modify native structures of soy proteins to increase their solubility and emulsification properties.

Protein functions are intrinsically linked to protein structures. Previously, highly sensitive processing technologies have been developed for effective modification of soybean proteins [6,7,8]. Among them, ultrasonic technology has been found to be a safe and environmentally friendly method of modifying proteins as it does not involve chemical treatments. This method leverages on the cavitation phenomenon at a frequency which is beyond the threshold of human hearing (>16 kHz) and is currently applied in food industry [9,10,11]. High-intensity ultrasound reduces the particle size of proteins. In soy protein isolates, it enhances the foaming properties, emulsion stability, and emulsifying activity of soy protein isolates or soybean glycinin (11S globulin) [5,10]. Hu et al. revealed that ultrasonic technology improved the fluid character and solubility of soy protein isolate dispersions [12]. This was attributed to the reduction of intermolecular interactions and partial unfolding. Also, O′Sullivan et al. [13] observed that ultrasound treatment reduced the particle size, improved solubility (pea and soy protein isolates) and the emulsification characteristics of egg white, bovine gelatin, and pea protein isolates.

However, in industrial food production, ultrasonication alone is time and energy-consuming; hence, less efficient. Researchers have proposed that combining ultrasound techniques with other modification techniques may overcome these limitations [9,13,14]. A recent study reported that ultrasound combined with transglutaminase treatment significantly improved the gelling properties of whey protein [15]. Ultrasound-assisted alkali or acid treatment accelerates enzymatic hydrolysis dissociation and protein solubility by unfolding and exposing hydrophobic groups. However, additional costs and safety risks limit its large-scale utilization [14,16].

Heating is a traditionally safe technique that improves protein functions. Of note, it induces multiple desirable structural changes in proteins [1]. Several studies have characterized the functional properties of protein-containing foods that have been structurally modified with thermal processing technologies [17,18]. However, the efficiency of protein modification using ultrasound in combination with heating has not been fully characterized. Ultrasound treatments increase the sensitivity of protein molecules to heating temperature. Based on the foregoing discussion, understanding the functional properties of soy proteins treated with ultrasound alone or in combination with heating is essential for its optimal industrial utility.

The 11S globulins are major storage proteins in soybeans accounting for nearly 40–60% of the total seed endosperm protein. They, therefore, contribute to the functional properties and nutritional quality of soybean proteins [3]. In this study, the effect of ultrasound alone or in combination with heating (50 and 60 °C) on the structural and functional properties of soy proteins was evaluated. Structural alterations were examined by intrinsic fluorescence spectroscopy, Sodium dodecyl sulfate -polyacrylamide gel electrophoresis (SDS–PAGE), ultraviolet (UV) absorption spectroscopy, and Fourier transform infrared (FTIR) spectroscopy. Size distribution and zeta-potential were evaluated using a particle size analyzer. The findings of this study show that ultrasound combined with heat treatment improves the functionality of 11S globulins-containing foods.

## 2. Results and Discussion

### 2.1. Structural Characterization

#### 2.1.1. SDS-PAGE Analysis

**Figure 1:**
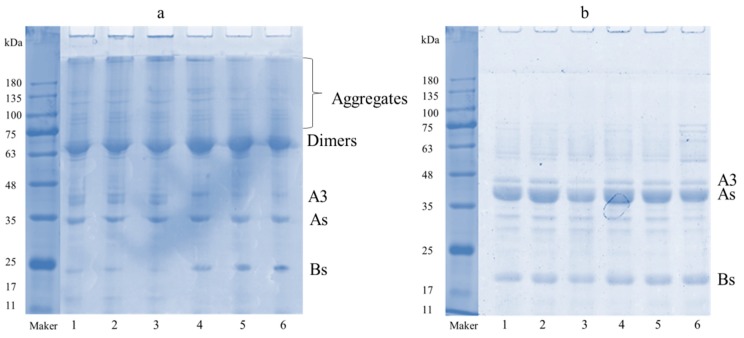
Non-reductive SDS-PAGE electrophoresis profile (**a**): 1–6 without 2-mercaptoethanol (2-ME) and SDS-PAGE electrophoresis profile (**b**): 1–6 with 2-ME. 1-Control, 2–50 °C, 3–60 °C, 4-Ultrasound, 5-Ultrasound-50 °C, 6-Ultrasound-60 °C.

Samples concurrently exposed to mild heating and ultrasound, mild heating or ultrasound alone were characterized by nonreductive SDS-PAGE and SDS-PAGE to analyze the disassociation-and-association behavior (Figure 1) [19]. Notably, the protein electrophoresis pattern was similar between the groups, suggesting that ultrasound and mild heating did not modify 11S globulin primary structure. This result is consistent with that reported previously [19,20,21]. Incremental increases in heating temperature caused more soluble aggregates lacking Bs subunits to form appeared above the separation gel (Figure 1a). This showed that high--temperature heating increased protein aggregation and decreased Bs subunits in the protein. Whereas ultrasound treatment reduced the formation of aggregates, ultrasound combined with mild heating completely prevented the formation of aggregates while increasing the Bs subunits content (Figure 1b). This suggests that the treatments disrupted the protein aggregates into small particles via the cavitation effects. Moreover, in the nonreductive SDS-PAGE analysis, ultrasound treatment increased the number of dimers formed by the proteins, indicating that the formation of aggregates induced by heating was due to noncovalent interactions (e.g., electrostatic and hydrophobic interaction) rather than covalent bonds (such as, intermolecular disulfide) between protein molecules. Figure 1b shows that addition of 2-ME did not induce dimers formation, indicating that heating caused dimers formation through disulfide bonds. The structural characteristics were further investigated with fluorescence spectra, UV spectra, and FTIR techniques.

#### 2.1.2. Fluorescence Spectra Analysis

**Figure 2:**
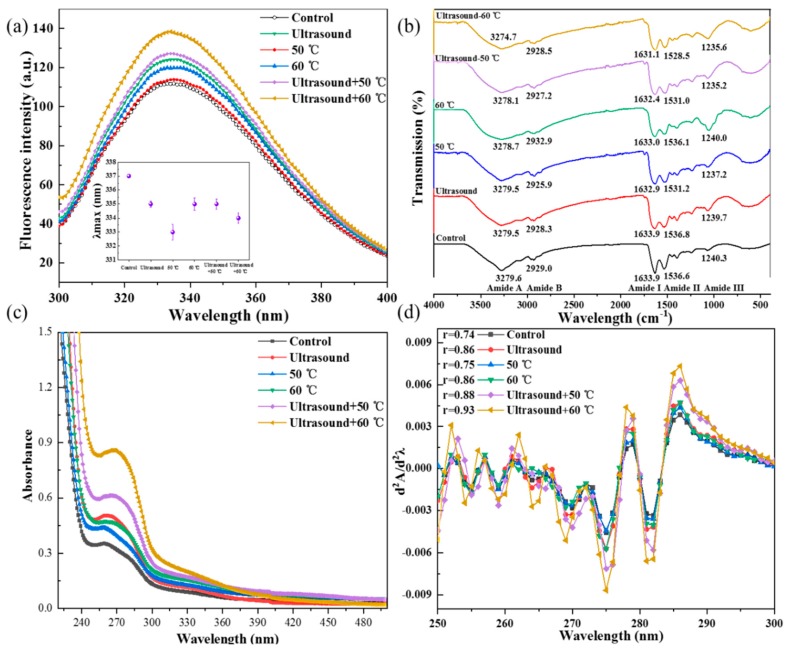
Effect of mild heating combined with ultrasound and ultrasound alone on the structure of 11S (**a**) Intrinsic fluorescence spectra, (**b**) Changes in FTIR, (**c**) UV spectra, (**d**) Second-derivative UV spectra.

Changes in the tertiary structure of 11S globulins after ultrasound treatments were investigated by fluorescence spectroscopy (Figure 2a). In the control samples, the maximum emission wavelength (λ_max_) was 337 nm. For 11S globulins treated with heating alone (50 and 60 °C), λ_max_ was not changed but the fluorescence intensity of these samples was higher than that of the control group. As shown in Figure 2a, the fluorescence intensity of 11S globulins treated at 60 °C was markedly higher than that of 11S globulins treated at 50 °C. With an incremental increase of the heating temperature, the protein unfold increased and more chromophores were exposed, which elevated the fluorescence intensity. The wavelength of protein samples exposed to ultrasound or ultrasound combined with heating (50 and 60 °C) showed a blue shift, accompanied by a marked increase in the fluorescence intensity. This indicated that these treatments disrupted the three-dimensional structure of 11S globulin protein, causing protein unfolding and shifting of hydrophobic groups in the protein towards a more hydrophobic environment (increased hydrophobicity) [1,10]. Similar blue shifts accompanied with an increase in fluorescence intensity were also reported in soybean protein isolates treated with ultrasound-assisted mild heating [1]. Wei et al. [22] stated that the blue shifts in the λ_max_ reflect a phenomenon where the previously exposed hydrophobic groups of soybean protein are buried into the interior of the modified protein.

#### 2.1.3. UV Spectra Analysis

The absorbance intensity of 11S globulins exposed to mild heating or ultrasound combined with mild heating increased significantly coupled with a redshift at the maximum absorbance wavelength (*p <* 0.05) compared with the control (Figure 2c). This may be ascribed to the exposure of buried hydrophobic groups and chromophores following structure unfolding [23]. The second-derivative UV spectroscopy is highly sensitive to alterations in the tertiary structure of protein molecules and can be used to delineate the contributions of three aromatic amino acid residues (Phe, Tyr, and Trp) [23]. In this study, the second-derivative spectrum of 11S globulin presented two positive peaks at 287 and 296 nm and two negative peaks at 284 and 292.5 nm (Figure 2d). Similar results were reported by Jiang et al. [24]. The peak at 270–285 nm regions and above 290 nm was attributed to the absorbance of Tyr, and Trp residues [23]. A continuous red shift of bands in the regions with Tyr and Trp residues after ultrasound combined with heating (50 and 60 °C) indicated that the protein underwent partial unfolding and more Tyr/Trp residues moved to the hydrophobic region [23]. The amplitude of the derivative spectral bands, obtained by calculating the ratio (r = a/b) of the two peak-to-trough values (Figure 2d), was used as a sensitive probe to predict the polarity of Tyr residues [25]. Samples exposed to ultrasound-assisted heating showed a slow increase in r values from 0.74 (control) to 0.75 (50 °C), 0.86 (60 °C), 0.88 (ultrasound-50 °C) to 0.93 (ultrasound-60 °C). This indicated that with an increase in energy input, more Tyr residues moved to nonpolar regions because of unfolding of the protein tertiary structure [25]. The second-derivative UV results were consistent with the fluorescence spectrum analysis, showing that ultrasound-assisted mild heating induced structural unfolding in 11S proteins. Jin et al. [23] noted that exposure of β-conglycinin to high pressure exhibited a similar redshift and a decrease in r value, which were attributed to the shifting of Tyr residues into the hydrophobic regions following protein unfolding.

#### 2.1.4. FTIR Analysis

FTIR is a method used to evaluate the secondary structure of proteins and polypeptides. In this technique, repeat subunits results in nine characteristic IR absorbance bands, marked as amide A, B, I, II, III, IV, V, VI, and VII [26] (Figure 2b).

In the present study, the FTIR of samples exposed to ultrasound alone or in combination with mild heating exhibited similar peak shapes but varying absorbance intensities (Figure 2b). The intensity of all absorbance peaks was higher in treated 11S globulins samples than in control samples, excluding amide A band, which corresponded to the stretching vibration of the OH groups and NH stretching from 3400 to 3440 cm^−1^. Once the CO-NH groups were joined by hydrogen bonds, the position moved to lower frequencies close to 3300 cm^−1^. The amide A band of the samples subjected to ultrasound-assisted mild heating was lower than that of the control samples, suggesting that more NH groups in samples were involved in hydrogen bonding [27]. There were more α-helix structures in the samples exposed to ultrasound-assisted mild heating than in control samples. The amide B peaks in the samples ranged from 2925.9 to 2932.9 cm^−1^, which corresponded to hydrophobic interactions caused by CH stretching [28] (Figure 2b).

The peaks ranging from 1633.9 to 1631.1 cm^−1^ corresponded to amide I (1600–1700 cm^−1^), assisted with the CO stretching vibrational frequency [26]. The absorbance peaks of the treated 11S globulins were lower compared to the control group11S globulins, indicating the formation of hydrogen bonds between NH and CO formed α-helix [27]. A similar phenomenon was observed in the amide II band of 11S globulin samples (1536.6 to 1528.5 cm^−1^), which was attributed to NH in-plane bending and the CN stretching vibrations. Similar results were obtained for amide A band. Treatment of 11S globulins formed amide III bands ranging from 1240.3 to 1235.6 cm^−1^, due to CN stretching, NH in-plane bending from amide linkages and CH_2_ wagging vibrations (Figure 2b).

The amide I and II absorbance and vibration frequencies are strongly and positively correlated with the secondary structure components [28]. Herein, the amide I and II peaks of 11S globulins exposed to ultrasound combined with mild heating had a lower frequency compared to the control samples, ultrasound alone, and mild heating alone, indicating loss of the higher structure due to combined treatment. The combined treatment of 11S globulins yielded fewer intermolecular crosslinks and higher flexibility, which matched with the electrophoresis pattern, intrinsic fluorescence, and UV spectrum results.

**Figure 3:**
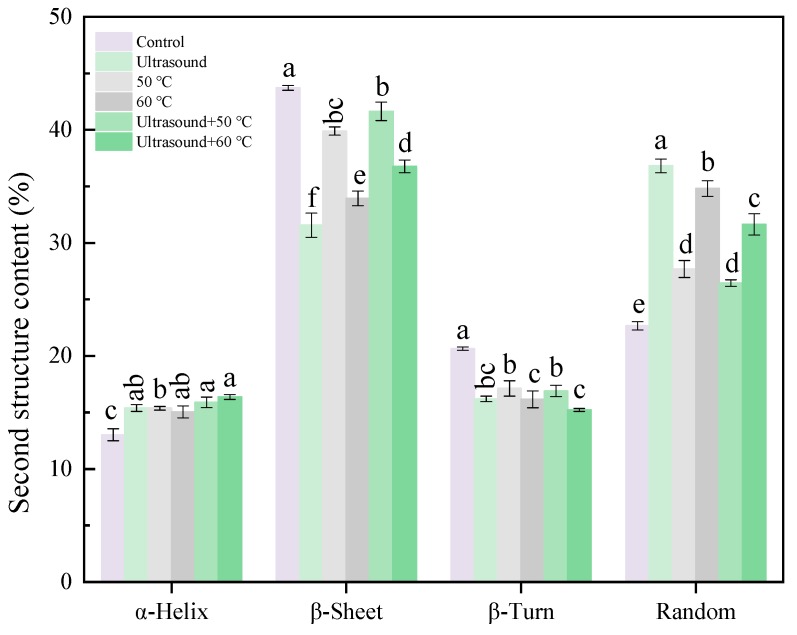
Secondary structure contents of 11S globulin after different treatments analyzed by curve-fitting. Note: Values with the same superscript letters are not significantly different (*p <* 0.05, RM ANOVA, Duncan test).

The curve-fitting method was employed to characterize alterations in secondary structure of 11S globulins. The random coil and α-helix were remarkably (*p <* 0.05) higher in the treated 11S globulins compared to the control samples (Figure 3). Moreover, mild heating or ultrasound treatments decreased β-sheet and β-turn content. Tang et al. [29] and Ren et al. [30] found that ultrasound treatment boosted the α-helix content. It has also been reported that ultrasound treatment increases the α-helix content in bovine serum albumin (BSA) aggregates and decreases β-sheet [31]. These results suggest that heating or ultrasound treatment disrupts and weakens the interactions between protein molecules. These findings are in agreement with the results of the intrinsic fluorescence spectrum and UV spectrum. Jin et al. [32] found that ultrasound treating decreased the α-helix content and increased the β-Sheet content in proteins. In contrast, Xiong et al. [33] found that ultrasound treatment did not change the secondary structure of ovalbumin. These discrepancies may be ascribed to protein-specific properties and ultrasound-treatment conditions. In the current study, the lowest β-sheet content and the highest random coil content were affected by ultrasound. However, mild heating combined with ultrasound treatment increased the β-Sheet content, which also led to the loss of random coil. The loss of random coil reflected re-aggregation of smallest particles into media particles, which matched the results of size distribution. 

### 2.2. Zeta Potential and Particle Size Distribution

**Figure 4:**
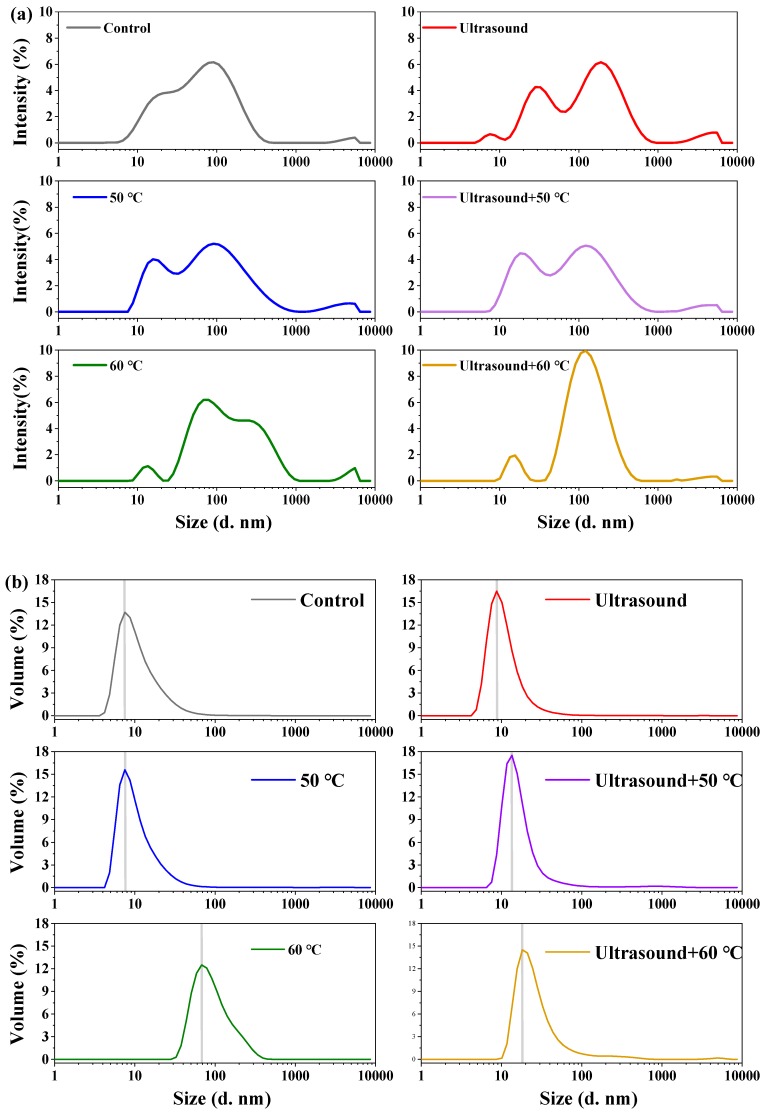

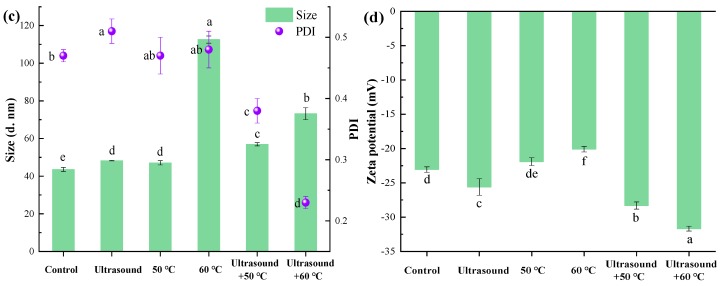
Zeta potential and particle size distribution of 11S (**a**): Intensity% distribution; (**b**): Volume% distribution; (**c**): Value of size and PDI; (**d**): Zeta potential) (*p <* 0.05, RM ANOVA, Duncan test).

The samples showed a broad particle distribution with the main peak of about 100 nm (Figure 4a). The particles size was negligible, being less than 100 nm (Figure 4b). The relationship between two identical populations was 1:1000 (*v/v*) in the volume distribution, and 1:1,000,000 in the intensity size distribution.

The average particle size of the control and treated 11S globulins was 8 nm, which corresponded to promoter homo trimeric of 11S globulin (9.5 nm × 9.5 nm × 4.0 nm) [34]. The particle size was larger in treated 11S globulins than in the control samples. Heating at 60 °C markedly increased the protein size by decreasing aggregation (Figure 4c), whereas ultrasound treatment decreased the particle size and increased the protein size. Ultrasound combined with mild heating decreased the particle size by disrupting aggregation. Heating increased the molecular weight which may enhance the probability of the particles being attacked by the cavitation effects. Indeed, the soluble aggregates at 10,000 nm sizes were broken into small particles. Morales et al. [5] observed that simultaneous ultrasound and heating treatment decreased the particle size of soybean proteins. However, the particle size of 11S globulins exposed to ultrasound heated at 50 °C was larger than those heated at 50 °C. Changes in protein particle sizes are influenced by collision and soluble aggregate formation through noncovalent interactions (e.g., hydrophobic interaction) [35]. Several studies found that ultrasound treatment enlarged the particle size of egg proteins and bovine serum albumin by increasing hydrophobic interactions [35,36,37]. Herein, the combined treatment reduced the particle size into medium-size, resulting in a smaller polydispersity index (PDI). A PDI value greater than 1 indicates that the sample has a broad size distribution. (Figure 4c). This suggests that the particle distribution range of 11S globulin dispersion was reduced and the dispersion of the 11S globulins particles in water was enhanced [38]. These findings are in agreement with those of FTIR, SDS-PAGE and emulsion analyses.

Zeta potential indicates the stability of a system. A higher zeta potential with stronger repulsive forces between protein particles inhibits the collision and aggregation. The dispersion and aggregation of 11S globulin solutions are determined on the basis of effective surface charge [31,35]. In the present study, all samples exhibited negative zeta potentials (Figure 4d), suggesting high density of negatively-charged amino acid residues on the protein surface. Notably, heating decreased the absolute zeta potential of 11S globulin but ultrasound combined with heating produced opposite effects. Similar results have been reported for BSA [31] and protein isolates from black beans [35]. Combined treatment increased the negative charge on the protein surface, indicating that solution stability was improved by the cavitation effects, which disrupted existing aggregates and strengthened electrostatic repulsions, hence inhibiting further aggregation. This enhanced the solubility of 11S globulin, improving emulsion properties.

### 2.3. Protein Flexibility Analysis

The flexibility of a protein structure regulates its surface activity and emulsification [39,40]. The molecular flexibility of a protein can be detected using the protease digestion method [41] and presented as the comparative motion of different structural intervals in proteins or the amino acid residue rearrangement rate in polypeptide chains [40]. Flexibilities of proteins performing the same catalytic activity seem to be about the same at their temperature optima. But rigid thermostable proteins reach the flexibility of thennolabile proteins at higher temperatures [40].

**Figure 5:**
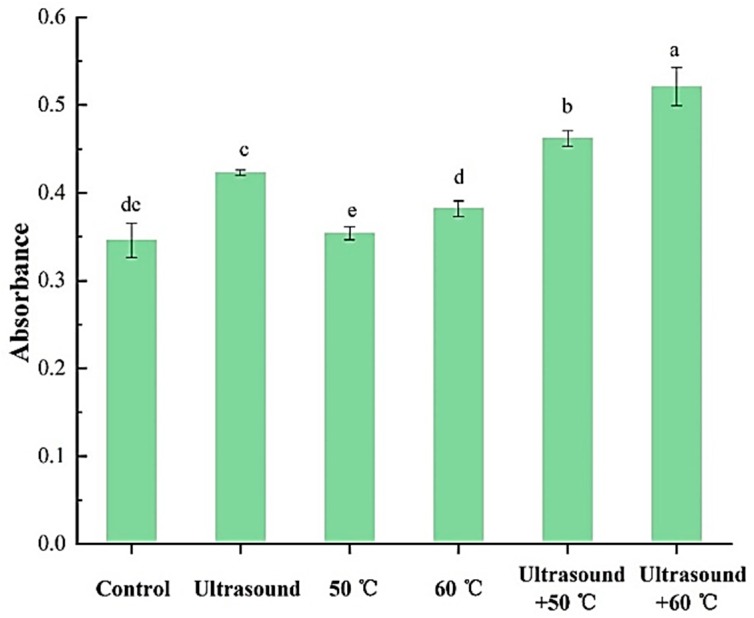
Effects of ultrasound with or without mild heating treatments on the flexibility of 11S globulin (*p <* 0.05, RM ANOVA, Duncan test).

The flexibility of 11S globulins subjected to ultrasound treatment alone or in combination with mild heating was higher than of the control group (Figure 5), indicating that heating unfolded 11S globulin protein molecules. This was basically achieved by cavitation and heat effects on the tertiary and quaternary structures of 11S globulins, which disrupt non-covalent bond cleavage, thus destroying the rigid structure inside the protein. As a result, the protein can be easily broken as the temperature increases [39]. Previous studies have suggested that the globulin sensitivity to protease digestion is closely associated with level of protein unfolding level [40].

### 2.4. Impact of Ultrasound Treatment on Emulsifying Properties of 11S Globulin

**Figure 6:**
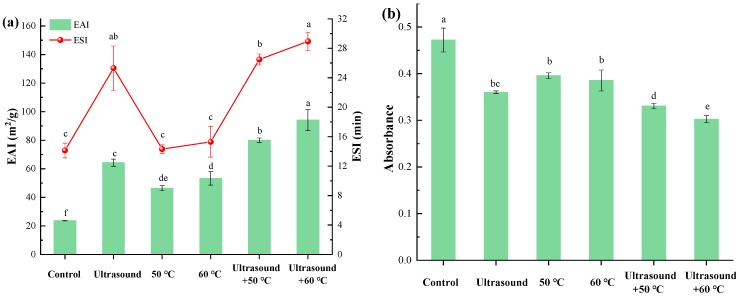
Effects of mild heating, mild heating combined with ultrasound and ultrasound treatments on emulsifying properties of 11S globulin (**a**) and free protein (**b**) (*p <* 0.05, RM ANOVA, Duncan test).

Our results show that mild heating, mild heating combined with ultrasound, and ultrasound treatments improved emulsifying activity index (EAI) (Figure 6a). This is because ultrasound treatments increased solubility, flexibility, and hydrophobicity of the protein by changing its structure. Thus, the adsorption rate at the oil-water interface was effectively improved (Figure 6b). The EAI values of ultrasound samples exhibited were higher value than that of those heated 11S globulin. This may be explained by the fact that ultrasound treatments increased flexibility and hydrophobicity, which improved protein molecule absorption at the water-oil interface and fewer free protein in the aqueous phase of emulsions. Similar phenomena have been observed in the emulsion properties of other proteins, such as peanut protein isolates [21], black bean protein isolates 40), faba bean (*Vicia faba* L.) protein, and myofibrillar proteins [20]. In this study, ultrasound-assisted mild heating improved EAI by triggering the formation of uniform-size protein nanoparticles (Figure 6a). Uniform-sized protein nanoparticles have been shown to enhance emulsion properties [42], making such proteins effective drug delivery systems and vaccine adjuvants [42]. Uniform-sized protein nanoparticles also improve the water holding capacity and gel strength of proteins [43].

The thermodynamic properties and stability of 11S globulin emulsions are influenced by flocculation, aggregation, and coalescence of emulsion droplets [20]. In this study, the changes in emulsion stability index (ESI) were similar to variations in EAI (Figure 6a) and were strongly enhanced by ultrasound or combined treatment. This is because ultrasound treatment increased the density charge on the protein surface, structural flexibility, hydrophobicity, and uniformity of particles sizes. Similar results have been reported in myofibrillar protein [20] and peanut protein isolates [21].

### 2.5. Optical Microscopy Analysis of Emulsions

**Figure 7:**
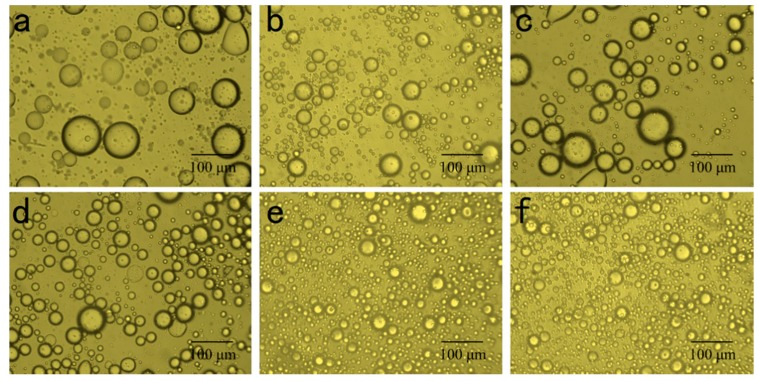
Optical photomicrographs of emulsions of 11S globulin exposed to mild heating (**c**) 50 °C, (**d**) 60 °C, mild heating combined with ultrasound (**e**) Ultrasound-50 °C; (**f**) Ultrasound-60 °C, (**b**) ultrasound alone and (**a**) untreated 11S globulin as control.

To further understand the relationship between the protein particle characteristics and emulsion properties, the micro-topography of the treated and untreated samples was examined at the same magnification (400×). The emulsion prepared from control samples (a) exhibited flocculation and had the largest droplets (Figure 7). This instability was ascribed to the poor protein flexibility and solubility, and the broad size particle distribution which decreases emulsification at the oil-water interface. Notably, mild heating (c,d), ultrasound treatments (b) or combined treatment (e,f) decreased the size of oil droplets and increased the uniformity of particles distribution. This phenomenon implies that the emulsifier was distributed on the oil-water interface, which improved the structural flexibility, increased the charge density and size uniformity of protein particles. 

## 3. Materials and Methods

### 3.1. Materials

Soybean meal devoid of fat (protein 51.2%) was obtained from Hexu Food Co., Ltd. (Heilongjiang, China). Soybean oil was procured from Beidahuang Grain Group Co., Ltd. (Harbin, China). 8-Anilino-1-naphthalenesulfonate (ANS), 5,5-dithio-bis (2-nitrobenzoic acid) (DTNB), n-hexane, sodium dodecyl sulphate and trypsin (16830 U/g) were obtained from Sigma (St. Louis, MO, USA). All the chemicals were used in the present study were of analytical grade quality.

### 3.2. Preparation of 11S Fraction

The alkaline extraction and acid precipitation technique described by Johnson et al. was used to isolate the 11S globulin fractions [44]. Briefly, defatted cold-pressed soybean meal was dispersed in Tris-HCl buffer (pH 8.5, 0.05 M) followed by vibration extraction at 50 °C for 1 h. The extract was centrifuged at 4400× *g* for 15 min. Afterwards, the pH of the extract solutions was adjusted to 6.4 using 2 M HCl and allowed to stand for 30 min at 25 °C to precipitate the proteins. The extracts were centrifuged at 4400× *g* for 15 min and dissolved in deionized water with a pH of 7.0 (adjusted using 2 M NaOH). Finally, the samples were freeze-dried.

### 3.3. Ultrasound and Mild Heating of 11S

The freeze-dried 11S globulins were solubilized to a concentration of 10 mg/mL (*w/v*), then treated with either ultrasonic or mild heating. For ultrasound-assisted mild heating, the samples were maintained in a water bath at 50 and 60 °C while being treated by ultrasonic energy at 20 kHz at 240 W for 30 min (NingBo Scientz Biotechnology Co., Ltd., Ningbo, China). For ultrasonic heating alone, samples were maintained at 25 °C while being treated with ultrasonic energy of 20 kHz at 240 W for 30 min. For mild-heating, the samples were maintained in a water bath with a temperature of 50 and 60 °C for 30 min. The control group samples were not exposed to any treatment.

### 3.4. Emulsifying Capacity Index

The ESI and EAI of protein samples were determined by dissolving the samples in phosphate-buffered solution (PBS, 10 mM, pH 7.2) to achieve a protein concentration of 50 mg/mL (*w/v*). Aliquots of 15 mL protein solution were homogenized with 5 mL soybean oil with an ultra-turrax at 10,142× *g* for 1 min. An amount of 50 µL of the emulsion was collected from the bottom of the homogenized emulsion immediately at 0 min and after 10 min, then diluted in 5 mL of 1 mg/mL (*w/v*) sodium dodecyl sulfate (SDS) solution. After vortexing for 5 s, the absorbance of the diluted emulsions was measured at 500 nm using a spectrophotometer [45]. The ESI and EAI were determined by Equations (1) and (2), respectively:

(1) $${\mathrm{EAI}\;(m}^{2}/g)\;=\;\frac{2\;\times \;{2.303\;\times \;A}_{0}\;\times \;\mathrm{DF}}{C\;\times \;(1\;-\;\theta )\;\times \;10000}$$

(2) $$\mathrm{ESI}\;(\min)\;=\;\frac{A_{0}}{A_{10}}\;\times \;10$$

where DF is the dilution factor, C is the initial concentration of protein (g/mL), θ is the volume fraction of oil in the sample, A_0_ is the absorbance immediately after emulsion formation, and A_10_ is the absorbance at 10 min following formation of emulsion.

### 3.5. Measurement of Protein Concentration in the Emulsion Solutions

The free protein content in the emulsion was quantified as described previously [46]. Samples were centrifuged at 10,142× *g* for 10 min and 0.2 mL of the solution was collected from the bottom layer and mixed with 5 mL G-250 and 0.8 mL distilled water. The protein concentration was determined with a UV–visible spectrophotometer (Lambda 365, Perkin Elmer Instruments Co., Ltd., Waltham, MA, USA).

### 3.6. Protein Flexibility

The molecular flexibility of the protein extract was evaluated as previously described [41]. Briefly, 250 µL of 1.0 mg/mL (*w/v*) trypsin solution (0.05 mol/L Tris-HCl buffer solution, pH 8.0) was mixed with 4 mL of 1.0 mg/mL (*w/v*) protein solution at 37 °C. After 5 min of reaction, 4 mL of 5 mg/mL trichloroacetic acid (TCA) was added to the reaction solution to terminate the enzymatic reaction and precipitate the protein. The solution was left to stand for 10 min before centrifugation at 2200× *g* for 15 min. Subsequently, protein flexibility was estimated by measuring the absorbance of supernatant at 280 nm. 

### 3.7. Measurement of Zeta-Potential and Size Distribution 

After treatment, samples were dispersed in PBS (10 mM, pH 7.2) at 1 mg/mL (*w/v*), and then filtered via a 0.45 μm cellulose membrane. The filtered samples were transferred into a cell then diluted 10 times using MilliQ water. Particle size and zeta-potential were calculated using a particle size analyzer (Zetasizer ZS-90, Malvern Instruments, Worcestershire, UK). 

### 3.8. SDS-PAGE

An SDS-PAGE analysis was performed according to the method published by Zhao et al. with some modifications [47]. The stacking gel and the resolving gel were 5% and 12% acrylamide, respectively. Protein samples (20 μL, 1.0 mg/mL) were mixed with loading buffer containing SDS and β-mercaptoethanol at 1:1 (*v/v*). Next, the solutions were boiled for 10 min and centrifuged. Then, 50 μL sample was loaded onto each lane of the SDS–PAGE gel.

Non-reductive gel electrophoresis without β-mercaptoethanol was performed similar to SDS-PAGE method.

### 3.9. UV Spectrum

The UV absorption capacity of the protein extract was determined as previously described by He et al. [48]. Briefly, we measured the UV spectra of the treated samples and controls (1.0 mg/mL (*w/v*) at 240 to 320 nm at an interval of 1.0 nm using Perkin Elmer LAMBDA 365 (Perkin Elmer Instruments Co., Ltd., Waltham, MA, USA). The measurements were taken at room temperature with PBS buffer (10 mM, pH 7.2) as a blank sample. The second-derivative UV spectra of control and treated protein samples were analyzed with Origin Pro (2017) software (Origin-Lab Co., Northampton, MA, USA).

### 3.10. Intrinsic Fluorescence Spectroscopy

The intrinsic fluorescence spectra of the protein extracts were obtained with a Hitachi F-7000 Fluorescence Spectrophotometer (Hitachi, Ltd., Tokyo, Japan). Samples were dissolved in PBS (10 mM, pH 7.2) at 0.1 mg/mL (*w/v*). The tertiary structure was examined using intrinsic fluorescence emission ranging from 300 to 400 nm with a 290 nm excitation under a slit width of 5 nm for both the excitation and emission slits).

### 3.11. FTIR 

The protein samples (1.0 mg) were assorted with KBr (100 mg) then grounded to a fine powder in an agate mortar under infrared light. The powder was pressed to form a pellet. The FTIR data were obtained at wavenumbers ranging from 400 to 4000 cm^−1^ over 32 scans with a 4 cm^−1^ resolution using a PerkinElmer two spectrum (Perkin Elmer Instruments Co., Ltd., Waltham, MA, USA). Data were analyzed with Omnic 8.2 software (Thermo Fisher Scientific Inc., Madison, WI, USA) and Peakfit 4.12 (Systat Software, San Jose, California, USA) [19].

### 3.12. Microscopic Examination of the Emulsion 

Samples were diluted 20 times and observed under 400× optical microscope with a XP-213 Transmittance and reverse polarized microscopy (Shanghai Precision Instrument Co., Ltd., Shanghai, China) equipped with a CCD camera.

### 3.13. Statistical Analysis

Data were analyzed and presented using SPSS 22.0 software (SPSS Inc., Chicago, IL, USA), Origin Pro (2017) software (Origin-Lab Co., Northampton, Massachusetts, USA), and Peakfit 4.12 (Systat Software, San Jose, CA, USA). Significant differences between groups were set at *p <* 0.05. All experiments were repeated at least thrice.

## 4. Conclusions

Herein, the structure and emulsifying properties of 11S globulin subjected to mild heating, mild heating combined with ultrasound, and ultrasound alone treatments were investigated. Our findings show that ultrasound or ultrasound combined with heating treatments disrupts the solubility and aggregation of proteins into smaller particles and partially unfold 11S globulin. These methods are therefore suitable for modifying the emulsifying properties of 11S globulin. This was demonstrated by the disappearance of aggregates in SDS-PAGE analysis and changes in intrinsic fluorescence. Second-derivative UV spectroscopy showed that ultrasound combined with heating increased hydrophobicity and α-helix as wells as reduced β-sheet and uniform-sized protein particles. As a result, the emulsifying activity index was increased 6.49-folds (from 23.73 to 154.10 m^2^/g) compared to the control group. Moreover, the structural flexibility and surface charge density of the protein were significantly enhanced, leading to the oil-water interface entirely being distributed by the emulsifier. This improved the emulsifying stability index from 14.12 to 40.93 min.
